# How efficient is translation in language testing? Deriving valid student vocabulary tests in Spanish (StuVoc1-Esp and StuVoc2-Esp) from established English tests

**DOI:** 10.3758/s13428-025-02708-0

**Published:** 2025-05-30

**Authors:** Beatriz Bermúdez-Margaretto, Marc Brysbaert

**Affiliations:** 1 Departamento de Psicología Básica, Psicobiología y Metodología de Las CC. del Compto., Universidad de Salamanca, Salamanca, Spain; 2 Instituto de Integración en La Comunidad (INICO), Universidad de Salamanca, Salamanca, Spain; 3 Department of Experimental Psychology, Ghent University, Ghent, Belgium

**Keywords:** Language testing, Vocabulary tests, Spanish, L1 language knowledge

## Abstract

This study examined the efficiency of item translation in a challenging language-testing situation. We created a Spanish translation of recently developed English vocabulary tests to assess word knowledge in Spanish-speaking students and highly educated adults, a group for whom it is a challenge to find words that some people know and others do not. The English tests were multiple-choice tests based on meaning recognition and consisted of a total of 150 items. From these, we were able to create two Spanish tests with 37 questions each. We constructed and validated the tests in two separate studies, including another established vocabulary test (Lextale-Esp, based on form recognition), general knowledge tests, and a test for reading comprehension. Two online studies with 161 and 196 participants confirmed that both vocabulary tests have reliability above .75 (.86 when combined) and correlate more highly with general knowledge and reading comprehension than Lextale-Esp. This shows that test translation is an efficient way to find useful items for language tests in different languages. All materials (including the general knowledge tests and the reading comprehension test) are freely available for research purposes.

## Introduction

Good tests are essential in language research. Such tests provide reliable and valid estimates of the skills they are supposed to measure. Reliability refers to the stability of the measurement (across items and time), validity to how well the test measures what it claims to measure. Unreliable measurements make it pointless to interpret individual differences in performance; invalid measurements lead to incorrect theories about the processes contributing to language processing.

Reliability was long neglected in experimental language research because researchers were only interested in differences between groups. For example, they compared a group of native speakers (L1) to a group of second-language speakers (L2), or they studied a group of bilinguals to compare L1 processing with L2 processing. The difference in mean performance between the groups/conditions was important, not whether the differences between participants in the groups/conditions were reliable. The situation changed when language researchers started to make use of individual differences to test theories of language processing (Brysbaert, 2024). Suddenly, they were confronted with the fact that participants scoring high on some items did not score high on equivalent items, or that participants who scored high at one time no longer scored high when tested anew some time later (Byers-Heinlein et al., 2021; Cunnings & Fujita, 2021; Hedge et al., 2018; Siegelman et al., 2017; Staub, 2021).

Because language proficiency is a central topic in second language processing, L2 researchers have invested more energy in creating good language tests than L1 researchers.^1^ This is particularly true for English as L2 (e.g., Peters et al., 2019; Schmitt et al., 2020; Webb & Nation, 2017) and also to some extent for French as L2 (Peters et al., 2019) and Spanish as L2 (Robles-García et al., 2024). Even in the L2 literature, Lemhöfer and Broersma (2012) noted that “Given the central role of proficiency— or vocabulary knowledge, in the case of single word processing—in L2 research, it is alarming how little consensus there is on how to measure it. Most bilingual studies within experimental psychology rely on participants’ self-ratings of proficiency and language background questionnaires as the only source of proficiency information” (p. 325). The authors subsequently presented the Lextale test to objectively measure language proficiency in English, Dutch, and German.

Language tests developed for L2 speakers are rarely useful for L1 speakers, because they are too easy. Whereas L2 speakers rarely master more than 8,000 words (Cobb, 2007; Laufer & Ravenhorst-Kalovski, 2010), L1 adults have an average vocabulary of over 40,000 lemmas (Brysbaert et al., 2016). As a result, nearly all L1 participants score close to maximum on L2 tests, and differences between individuals rarely have reliability above 0.5. This is likely why Lemhöfer and Broersma (2012) wrote (italics added): “In psycholinguistic studies involving L2 speakers, however, a problem arises *that is virtually absent in monolingual research*—namely, the enormous variability of the participants’ proficiency levels and, in particular, of levels of vocabulary size, even within learner groups exposed to relatively homogeneous learning conditions” (p. 215).

A look at the psychometric literature reveals that Lemhöfer and Broersma’s (2012) claim of a lack of variability in L1 language knowledge is unfounded. Native speakers do vary in their knowledge of the native language. Indeed, vocabulary tests have been a core element of intelligence tests since their initiation in the early twentieth century (Anderson & Freebody, 1981). Terman et al. (1918) already reported a correlation of 0.91 between the entire Stanford Binet Scale and a vocabulary test in primary school children. On this basis, he suggested that the vocabulary measure alone constituted a good estimate of performance on the entire scale and thus could be used as a short measure of intelligence. Vocabulary size also correlates highly with reading and listening comprehension (Cromley & Azevedo, 2007; Mezynski, 1983; Verhoeven & Perfetti, 2011). It is used to test theories of language processing (Perfetti & Hart, 2002), measure the effects of differences in language exposure (Lee et al., 2019; Taylor et al., 2018), investigate age differences in language skills (Laumann Long et al., 2000; Verhaeghen, 2003), and examine the consequences of various life events (Brewster et al., 2022; Park et al., 2001). Therefore, vocabulary tests are an essential tool in many research settings.

Lemhöfer and Broersma (2012) were correct, however, that usable language tests for adult L1 speakers are rare, especially tests for students and highly educated adults, populations used in much language research. Vermeiren et al. (2023) noted that even in English (the language with the most resources), there were no validated, freely available vocabulary tests for L1 adult speakers. There were a number of commercial tests, which could be used by researchers, but these have limitations. The first is that they are not available to everyone (and cannot be forced on researchers by reviewers and editors). Because of copyright restrictions, researchers must purchase response forms if they want to use the tests and publish the results, so these tests are only available to those who can afford them. Second, the tests cannot be improved because they must be used as published. Finally, translating the tests without the publisher's permission is not allowed. This makes it impossible to create a set of parallel tests in different languages, unless the publishers do it.

To remediate the situation, Vermeiren et al. (2023) decided to develop a vocabulary test themselves. Starting from Nation’s (2020) Vocabulary Size Test, they selected a set of 100 items that looked useful for L1 students (Nation’s test was originally created for English L2 speakers). Items consisted of a visually presented target word, together with four response alternatives from which participants had to select the one closest in meaning to the target word. The items were tested on a group of 200 participants, but turned out to have low reliability and to measure more than one factor, forcing Vermeiren et al. (2023) to create and validate new items.

In the end, it took Vermeiren et al. (2023) five studies (and nearly 1,000 participants) to create a vocabulary test for English students, which had reliability greater than 0.8, loaded on a single factor, and demonstrated a correlation of greater than 0.3 with reading comprehension and general knowledge. The reason it was so difficult to find good items is that word frequency is not a useful selection criterion for people who know over 40,000 words. Nearly all unknown words are low in frequency, and it is very difficult to predict which of these words will be known by 40%, 60%, or 80% of the population (the type of words you need in order to find a reliable difference between low and high performers). Vermeiren et al. (2023) were helped because they had information about word prevalence (Brysbaert et al., 2019) and age of acquisition (Kuperman et al., 2012) for thousands of English words, but even then it took them several attempts to develop a valid test.

**Fig. 1:**
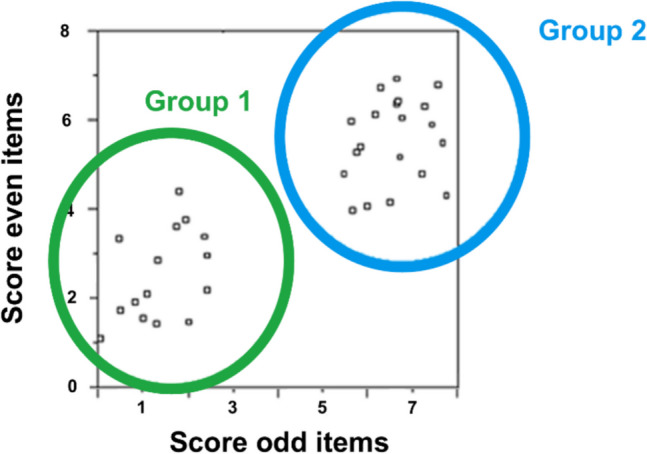
The problem of range restriction illustrated with split-half reliability. *Note*. Test scores of participants were calculated on the odd (*x*-axis of the figure) and even items separately (*y*-axis of the figure) and correlated with each other (split-half correlation). The correlation is good across the entire range of participants (*r* =.86) but is much smaller when tested for the low-performing group (*r* =.46) or the high-performing group (*r* =.23) alone

The search by Vermeiren et al. (2023) was even more difficult because they were testing a homogeneous group of undergraduate students. The variability in such a group is smaller than in the population at large, making it harder to find words that differentiate between very proficient students and less proficient students. The problem with homogeneous groups is called range restriction (Sackett & Yang, 2000) and is illustrated in Fig. 1. Although the correlation in Fig. 1 is good across the entire range of participants, it decreases considerably when tested for the high-performing or the low-performing group alone. To achieve high correlations within each group, items must be found that are group-specific.

Given the difficulties that Vermeiren et al. (2023) encountered in creating a good vocabulary test for students in higher education, an interesting question arises as to how much effort can be saved by using item translations to create a similar test in another language. Vermeiren et al. (2023) could make use of a wealth of information available only for English words (word frequency, word prevalence, word age ratings, etc.). Thus, creating a good test in a non-English language from scratch is even more daunting.

Test translation is often used to create a test in a new language, but less common in language research out of concern that findings with stimuli in one language may not generalize to stimuli in another language. An exception is the translations/adaptations of the Peabody Picture Vocabulary Test (Dunn, 1959). In this test, participants hear words and must choose a picture from four alternatives to go with the target word. Also, some widely used IQ tests including a vocabulary test are available in many languages. Arguably the best known example is the Wechsler tests.

In addition to the translation of commercial tests by publishers, research-based language tests are also sometimes translated and made available. For example, Kotowicz et al. (2021) translated and edited the British Sign Language Receptive Skills Test into Polish. The procedure took eight steps, three of which involved collecting pilot data from participants in the target population.

The translations discussed so far involved tests for the general population. As noted, creating a test for high-performing adults involves extra challenges due to range restriction. In the present study, we examined how much savings could be achieved by starting from an established test. In the worst-case scenario, no savings would be gained and the same number of studies would be needed. In the best-case scenario, the translated materials would be good, without the need for further testing.

To answer the question as to the efficiency of the translation approach (in terms of time and costs needed to achieve a reliable and valid test), we translated the English tests of Vermeiren et al. (2023) into Spanish. Spanish is one of the main languages in the world (in fact the second-most commonly spoken native language in the world, after Mandarin, with around 400 million speakers, according to Ethnologue, 2022), but to our knowledge has only one free vocabulary test for undergraduate students: Lextale-Esp, developed by Izura et al. (2014). It consists of a random list of 60 words and 30 nonwords. Participants are asked to indicate which words they know and are told that not all stimuli are existing words. They are informed that they will be penalized if they say yes to “words” that do not exist in the language (see also below). The test demonstrated a correlation of 0.82 with self-assessed Spanish proficiency. Ferré and Brysbaert (2017) subsequently showed that the test can be used with L1 undergraduate students, because Catalan–Spanish bilinguals with Spanish as the dominant language had higher scores than proficient Catalan–Spanish bilinguals with Catalan as the dominant language. No further validation studies were reported.

It would be beneficial to have more than one vocabulary test in Spanish (and other languages) because this allows researchers to investigate convergent validity: to what extent various tests measure the same construct (vocabulary knowledge). As for the yes/no word decision format used in Lextale-Esp (see Fig. 5 for an example of an item in this test), there is conflicting evidence about how well it predicts language proficiency (e.g., reading comprehension). Some researchers report good evidence (Harrington and Carey, 2009; Harsch & Hartig, 2016; Lemhöfer & Broersma, 2012; Mochida & Harrington, 2006; Nakata et al., 2020; Siegelman et al., 2024). Others, however, observe rather low correlations with language understanding (Cromheecke & Brysbaert, 2022; Eyckmans, 2004; McLean et al., 2020; Zhang & Zhang, 2020). This may be because participants are only required to indicate whether they recognize a word. Nothing is asked about the word’s meaning. A new Spanish test with multiple-choice meaning-related questions would be informative for this discussion, as it would tell us how well it converges with the Lextale format in a new language.

Altogether, our study had four research purposes. First, we wanted to develop a good (reliable and valid) Spanish vocabulary test for students and educated adults (namely, the Spanish Student Vocabulary Test or StuVoc-Esp), based on meaning recognition rather than form recognition (as in Lextale-Esp). Second, we aimed to determine the benefits of item translation as a general strategy for developing valid tests for languages with few resources. Third, we conducted a rigorous validation check for the new StuVoc-Esp tests. Fourth, we wanted to know how well StuVoc-Esp would correlate with Lextale-Esp and how well each test would predict the validation criteria. Because we also had to develop good validation tests, a fifth possible benefit of our research was the availability of a validated Spanish general knowledge test and reading comprehension test.

In Study 1, we discuss item translation, item selection, and first validation. In Study 2, we examine whether the findings from Study 1 can be replicated in a cross-validation study.

## Study 1

In Study 1, we translated the English stimuli of Vermeiren et al. (2023) into Spanish. To see how useful the translated items would be for vocabulary assessment in Spanish, in addition to calculating the reliability of the test, we also correlated the obtained scores with performance on Lextale-Esp (Izura et al., 2014), a general knowledge test, and a reading comprehension test.

Lextale-Esp was developed to measure Spanish vocabulary and, therefore, should correlate well with the new vocabulary test if both measure the same skill (word knowledge).

Vocabulary knowledge and general knowledge usually correlate well and together measure so-called crystallized intelligence, declarative knowledge stored in long-term memory (Cattell, 1943, Cattell, 1971). Therefore, a test of general knowledge is a good validation criterion to test how well the vocabulary test measures crystallized intelligence.

Finally, vocabulary tests are often used to predict reading comprehension (Andrews et al., 2020; Goring et al., 2021; Lervåg & Aukrust, 2010; Oakhill & Cain, 2012; Ouellette, 2006; Proctor et al., 2012; Roth et al., 2002; Siegelman et al., 2024; Tannenbaum et al., 2006; Yildirim et al., 2011) and language comprehension in general (Gottardo et al., 2018). Thus, adding a reading comprehension test provides us with more information about the value of the new vocabulary test.

The general knowledge test and reading comprehension test were from Vermeiren et al. (2023) as well. They were translated from English into Spanish.

### Method

#### Participants

A total of 237 participants (144 female, 116 male, and 7 indicating other gender; mean age = 20.25 years) took part in the study. With the exception of a small initial batch (*n* = 15, 6 female, mean age = 32.26, range = 24–48) who only completed the vocabulary tests, participants were recruited through Prolific services, with the following criteria: age range 18–21 years, native speaker of Spanish, and fluent speaker in this language. Time for completion of the study varied among participants, with an average of 40 min; participants were paid £5.50. Participants were excluded from the study if they showed no commitment when performing the tests (i.e., inaccurate response to more than one of the three easy control words included in the vocabulary tests, *n* = 3) or when criteria were not fulfilled (*n* = 3 reporting that they were non-native Spanish Speakers), leaving data from 231 participants for further analysis (mean age = 20.23, range = 18–48; 109 female, 115 male, 7 other).

The country of residence was mainly Spanish-speaking (136 in Mexico, 67 in Spain, 13 in Chile), except for seven participants from the United States, three from Germany, one from Canada, one from Iceland, and one from the Netherlands. Among participants, 181 were students, 18 were employed, and 32 were both studying and working. With regard to the highest education level achieved at the time of testing, 125 participants reported a high school degree, whereas 106 reported a higher level (53 had a professional bachelor’s degree, 45 had an academic bachelor’s degree, 7 had master’s degree, and 1 had a doctorate degree). The majority of the 213 student participants (*n* = 200) were studying in a post-secondary, non-compulsory education program. Most participants (*n* = 221) reported speaking languages other than Spanish (215 English, 45 French, 25 German, 8 Chinese, 3 Basque, 19 Catalan, 5 Galician and 19 other languages).

The number of participants was set to greater than 200, because we hoped to analyze the data of at least 200 participants. This number reduces the 95% confidence interval around the obtained correlations to 0.15, so that we would have rather stable estimates (see Brysbaert, 2024, or Vermeiren et al., 2023, for more information).

#### Materials

All materials can be found at https://osf.io/c8n2x/. All materials from Vermeiren et al. (StuVoc, reading comprehension test, general knowledge test) were translated into Spanish by the first author, who is a native speaker of Spanish and a fluent speaker of English as L2. Special care was taken to match the difficulty of the tests.^2^

#### Vocabulary test (StuVoc-Esp)

An interesting aspect of Vermeiren et al.’s (2023) work is that they published three English student vocabulary tests with 50 items each and four response options (StuVoc-Eng1, StuVoc-Eng2, and StuVoc-Eng3). The first two tests are equivalent; the last one is easier (and was too easy for English-speaking students in Vermeiren et al.). This provided us with a large and rich stimulus set to start from. Because translating a test inevitably involves the loss of items due to language and cultural differences, all words were considered candidates for translation, even though the third test was too easy for English students.

The stimuli (150 target words and response alternatives) were translated into Spanish. For 4/150 target words this was not possible because the English word did not have a translation in Spanish or it was translated by an easy-to-understand Spanish compound word. Very often, several translations were possible because the English word had near synonyms which also existed in Spanish. An obvious error to avoid for such words is translating a difficult English word into an easy Spanish word (e.g., translate baneful to *funesto*, which translates back to disastrous). To find the best translation, we were helped by the fact that both English and Spanish have word prevalence values (Aguasvivas et al., 2018; Brysbaert et al., 2019). Word prevalence refers to the percentage of people reporting that they know the word in a yes/no form recognition task (i.e., the format used in Lextale). This made it possible to match words on word prevalence in addition to word frequency. In addition to the target words (and a short uninformative sentence in which the target word was used), the four answer alternatives were also translated. If necessary, the wording of the alternatives was adjusted. The final translated version of the vocabulary test, including all response options and correct alternatives, can be found in the supplementary materials at https://osf.io/c8n2x/ (folder: Tests available for research).

**Fig. 2:**
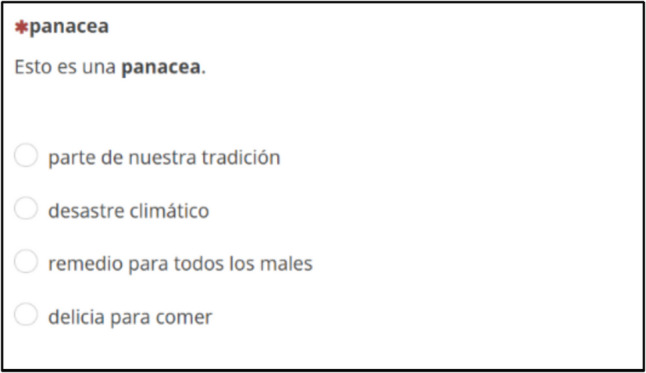
Format of the items used in the vocabulary test. *Note.* The target word *panacea* is presented, followed by the sentence in which the word is used (This is a panacea), and four response alternatives to choose from (part of our tradition, climate disaster, remedy for all ills (correct), and delight to eat)

Figure 2 gives an example of an item included in the vocabulary test. A target word is presented, followed by a short (2–6 words) uninformative sentence in which the target word was included (only giving information about the part of speech of the target word). Next followed four response alternatives (only one of which was correct), from which participants had to select the one closest in meaning to the target word. If participants did not know the target word, they were asked to guess the correct meaning (all items required a response).

In addition to the 146 words selected from the English StuVoc tests (48 words from StuVoc1, 49 from StuVoc2, and 49 from StuVoc3), nine control items were created. These consisted of high-frequency and high-prevalence Spanish target words that should be known to all speakers of Spanish. They were included (three per subset) to test whether the participants paid attention to the task. As indicated under the “Participants” section, we excluded participants who made more than one error on these control items. All in all, a total of 155 items were created for the Spanish vocabulary test, divided into three lists with 51, 52, and 52 words.

#### General knowledge test

**Fig. 3:**
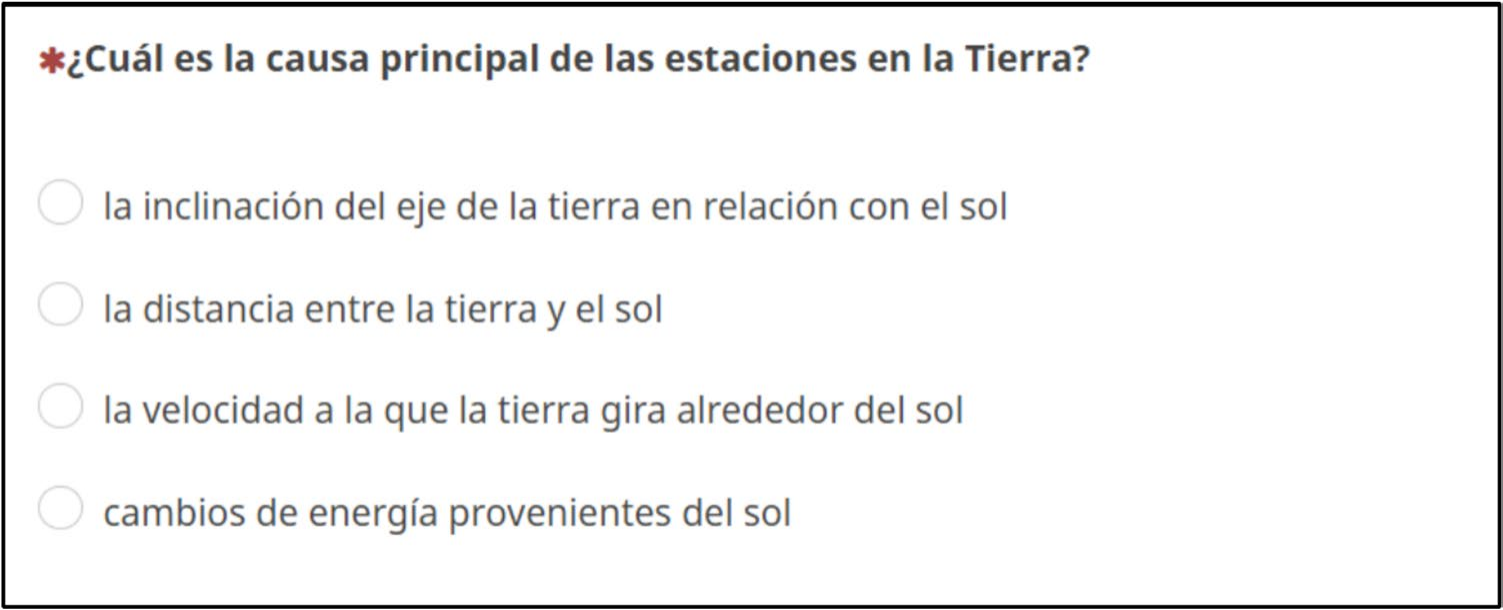
Example of an item presented in the general knowledge test**.** *Note*. A target sentence is presented (What is the main cause of seasons on Earth?) together with four response options: the tilt of the earth’s axis in relation to the sun (correct), the distance between the earth and the sun, the speed the earth rotates, changes in energy coming from the sun)

Vermeiren et al. (2023) compiled a general knowledge test with 65 items, some of them from the 1,000-question Spanish general knowledge database (Buades-Sitjar et al., 2021). These were short questions with four response alternatives covering a wide range of topics (geography, history, arts, technology, etc.). Importantly, the test was designed in such a way that it was relatively free of vocabulary knowledge. So, for example, a question would not be “What do you call a horse-like animal with black stripes?” but “How many wonders were there in the ancient world?” Fig. 3 shows an example of an item. The language of the test was carefully checked (also with Google back-translation), and several words were adapted if necessary during the translation process.

#### Reading comprehension test

**Fig. 4:**
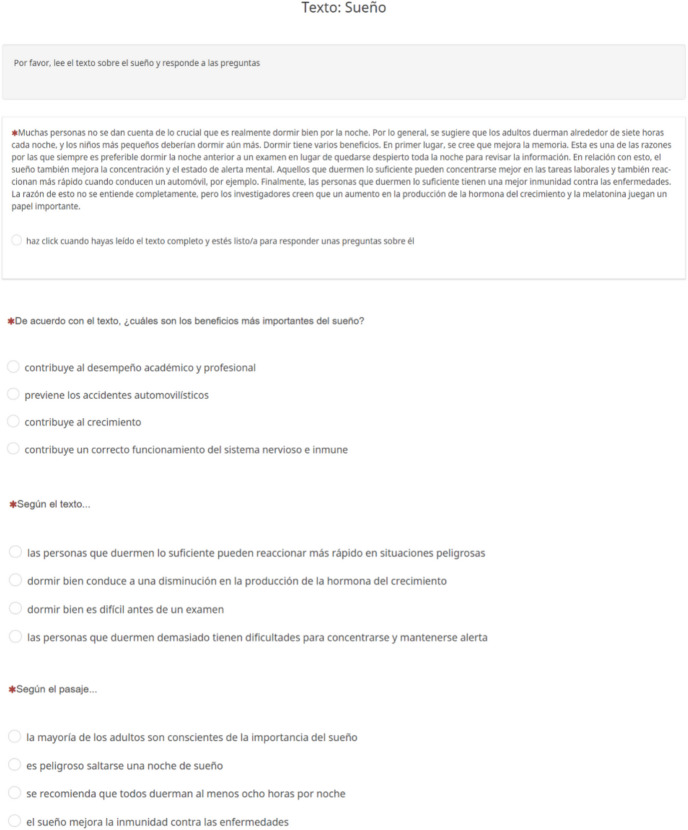
Example of an item presented in the reading comprehension test. *Note*. Questions were presented one after the other after the text had been read (and was no longer available). The original English version can be also found in the supplementary materials at https://osf.io/c8n2x/ (folder: Tests available for research)

Vermeiren et al. (2023) created a reading comprehension test consisting of 14 short expository texts (around 150 words each), based on Kuperman et al. (2022). Each text was followed by three multiple-choice questions with four response alternatives, to be answered after reading the text (i.e., the text was no longer visible when participants answered the questions). The texts were Wikipedia-type entries about different topics such as geography, technology, and life sciences (e.g., the Amazon Rainforest, the use of smartphones, or the need for sleep). Importantly, the texts described relatively unknown facts unlikely to be familiar to the participants, thus avoiding answers based on general knowledge (crystallized intelligence) rather than reading comprehension. Figure 4 shows an example of an item. All materials were carefully translated into Spanish and adapted if needed. Questions with poor performance in Vermeiren et al.’s (2022) test were rephrased or reformulated in an attempt to improve the reliability of the Spanish test (reliability of Vermeiren et al.’s test was ICC = 0.65).

#### Lextale-Esp

**Fig. 5:**
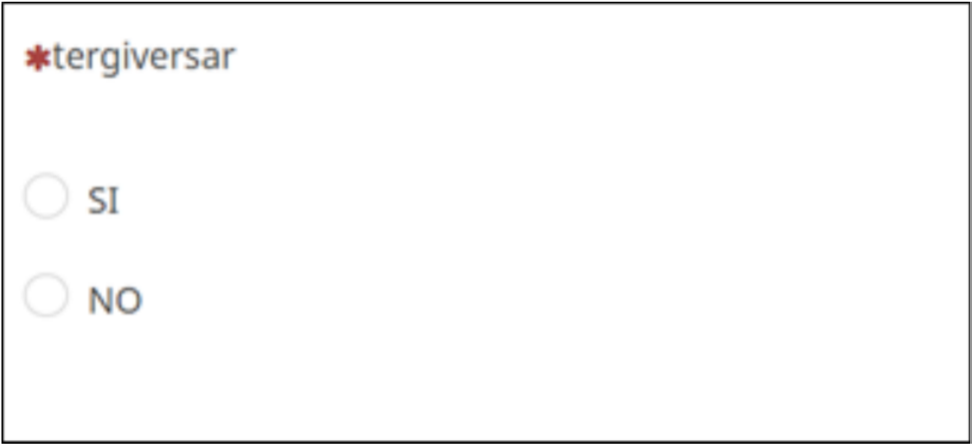
Example of an item presented in the Lextale-Esp. *Note*. A target word presented in the Lextale test (*tergiversar*, for which the closest English translations are “distort” and “misrepresent”), together with yes (SI) and no (NO) response options

The Spanish Lextale (Izura et al., 2014) was included as another vocabulary test of Spanish. This is a lexical decision task (yes/no answer) consisting of 90 items (60 Spanish words and 30 pseudowords). The presentation format of the published test was maintained, such as warning participants about the presence of nonword foils. The only exception was that both words and pseudowords required a response (i.e., both yes/no responses, whereas only yes responses to the words were required in the original test). See Fig. 5 for an example of the Lextale-Esp item presentation in the present study.

### Procedure

The different tests (new vocabulary test, general knowledge test, reading comprehension test, Lextale-Esp) were implemented in LimeSurvey (https://www.limesurvey.org/). Participants were recruited via Prolific (a company providing access to paid participants, https://app.prolific.co/) and tested online without the presence of a researcher. Participants were informed about the importance of completing all tests and taking each test seriously, as otherwise the data could not be used for scientific purposes. A reminder asking participants to take the study seriously, complete all tasks attentively, and answer all items was inserted again in the instructions provided with each test.

During the study, participants were first introduced to the goals and a general description of the study (including information about the number of tests, estimation of the time needed, and the payment they would receive). Participants were informed that the study could be done in 1 h, but that time was not restricted, so they could use more time if desired. Before starting the study, participants were asked to provide their explicit consent for participation and sharing anonymized data for research purposes.

The study started with six questions about demographic characteristics (age, gender, occupation, education level, native language, other languages spoken). The tests were then presented in the following order: Lextale-Esp, the 155 vocabulary items in three separate parts, the reading comprehension test, and the general knowledge test. The tests were presented as separate tests so that participants could take a short break in between if needed. All items and alternative answers were randomized within a test (with the exception of the reading comprehension test, where texts were randomized but the same order of questions had to be used because of technical constraints related to LimeSurvey). Responses were required to all items of the tests presented (i.e., participants could not skip items unless they stopped participation). The surveys used in the study can be found at https://osf.io/c8n2x/ (folder: LimeSurvey files).

### Data trimming and analyses

The time spent by each participant in completing each test was automatically recorded by LimeSurvey during data collection, and the accuracy of the response was automatically coded as 0 or 1 for each item. The average time was calculated for each test, and responses with durations deviating ≥ 2.5 *SD*s below or above the average time or with performance close to chance level were discarded before analyses started. This represented 3.4% of the data in the vocabulary tests, 7.79% in Lextale-Esp, and 5.8% in the general knowledge test.

For the reading comprehension test, words read per minute were calculated per text. Texts with reading rates lower than 100 words per minute and higher than 668 words per minute were considered outliers, because they are 2.5 times slower/faster than usual reading speed (Kuperman et al., 2021). Slow reading rates are characteristic of text studying, fast reading rates of text scanning (Brysbaert, 2019). This resulted in the loss of 16.1% of text data (on a total of 231 × 14 = 3,234 texts read). Altogether, only 207 out of 231 participants could be included in the analysis of the vocabulary tests, and 161 completed all tests correctly so that they could be included in the validation analysis.

Analyses were run with R software. In addition to descriptive statistics, we ran reliability analysis, factor analysis, and item analysis. For reliability calculations, the R package psych (Revelle, 2021) was used to calculate omega total and intraclass correlation ICC2k. The former was selected as the best index for estimating correlations between tests. The latter was chosen as an informative lower limit, based on the assumption that the questions were a random sample from a homogeneous population. It provides an estimate of the reliability that can be expected if a comparable set of questions is created (see Flora, 2020, for a review of the reliability measures that can be calculated). Values between 0.75 and 0.9 are typically considered indicative of good reliability, values less than 0.5 indicate poor reliability, and values above 0.90 suggest excellent reliability (Koo & Li, 2016). We aimed for reliability above 0.75, which is considered good for low-stakes tests where administration time is an important selection criterion.^3^

Factor analysis and item selection were carried out to ensure that different assumptions for good test construction were met, including that a single factor was measured, items differed in difficulty, item errors were uncorrelated, and all items had good discrimination power (Brysbaert, 2024). These criteria were used to select good items. Importantly, everything was done before we correlated test performance with the criterion variables, so that the findings could be generalized to future studies (cross-validation). Factor analysis was run with the psych package, based on tetrachoric correlations (Revelle, 2021). Regarding item analysis, we used multidimensional item response theory (IRT) analysis with the R package mirt (Chalmers, 2012), to detect and exclude items with suboptimal performance (those with poor discrimination) and to select items with well-spaced difficulty levels for each test.

Finally, performance obtained across all tests was correlated using Pearson correlation analysis. In this case, *r* scores between 0.5 and 1 were considered as indicative of strong correlation. All data and R code can be found at https://osf.io/c8n2x/files/; folder Study 1).

## Results

### Vocabulary test StuVoc

#### Descriptive statistics

Descriptive analysis showed that performance on the three subsets of the vocabulary test was largely equal (test 1: mean = 36.6, *SD* = 6.28, min = 18, max = 47/48, *N* = 216; test 2: mean = 37.5, *SD* = 5.69, min = 18, max = 48/49, *N* = 231; test 3: mean = 38.8, *SD* = 3.68, min = 25, max = 46/46, *N* = 218). This means that the accuracy difference between test 3 and the other two tests observed by Vermeiren et al. (2023) was less present in the translations. However, the standard deviation of test 3 was smaller than that of test 1 and test 2, indicating that the items came from a smaller range of difficulty levels than those of the other tests.

#### Reliability analysis

Some items were answered correctly by all participants and had to be eliminated because they showed no variability (test 1: *requisito* (requisite), *amuleto* (amulet), *ejemplificar* (instantiate); test 2: *laberinto* (maze), *huérfano* (unchin), *mugre* (muck); test 3: *ambigüedad* (ambiguity), *optimista* (upbeat). Reliability analysis conducted on the remaining items indicated good to excellent reliability: test 1: omega total = 0.85, ICC2k = 0.80; test 2: omega total = 0.82, ICC2k = 0.76; test 3: omega total = 0.70, ICC2k = 0.61).

#### Factor analysis and item evaluation

A parallel factor analysis based on tetrachoric correlations (Revelle, 2021) indicated that each test measured more than one factor and that item pruning was indicated. Single-factor IRT analysis with the R package mirt (Chalmers, 2012) was used to determine which items were not performing well within each subtest.

#### Item selection for two StuVoc-Esp tests

Although the translations of each StuVoc test by Vermeiren et al. (2023) performed well in terms of reliability, we decided to see whether further optimization of the Spanish test was possible. To achieve this, we combined the three tests into one dataset and selected items based on their discrimination value in the IRT analysis and on a good spread of item difficulty. We also checked whether there were major differences between participants from Latin America and Spain in word knowledge (making additional use of the prevalence figures published by Aguasvivas et al., 2018).^4^ Finally, we took into account that most researchers looking to use a vocabulary test as part of a study want the vocabulary test to be reasonably short (5–10 min).

In the end, we decided that we could select two good parallel tests with 37 items each. Table 1 provides information for each test and the two tests combined (in case a researcher needs a more precise test). The correlation between the tests was *r* = 0.74 (*n* = 207)

**Table 1:** Information for the two tests with 37 selected items and their combination (*n* = 207)

	Mean (*SD*)	Min	Max	Omega total	ICCk2
StuVoc-Esp1	30.0 (4.35)	14	37	0.81	0.73
StuVoc-Esp2	29.8 (4.02)	16	37	0.77	0.68
Combined	59.8 (7.80)	30	73	0.88	0.83

### General knowledge test

#### Descriptive analysis and reliability analysis

After exclusion of the participants who did not complete the test and participants responding at random (less than 18 correct responses on a total of 65), the mean number of correct answers on the general knowledge test was 40.6/65 (*SD* = 7.21, min = 17, max = 59/65, *N* = 193). Reliability analysis conducted on the full set of 65 items showed good reliability (omega total = 0.79, ICC2k = 0.74).

#### Item evaluation

A single-factor IRT analysis was run to see which items were not performing well. Ten items with low factor loadings (< 0.2) and poor discrimination curves were identified. Some of these items asked for information better known in English-speaking countries

### Reading comprehension test

#### Descriptive analysis and reliability analysis

Mean accuracy was used instead of sum scores to deal with the missing observations (see the texts with too fast or too slow reading rates). Accuracy was 0.71 (*SD* = 0.13, min = 0.28, max = 1.00, *N* = 199). Reliability analysis showed good reliability (omega total = 0.77; ICC2k = 0.70). The mean reading rate was 228 words per minute (*SD* = 97.21, min = 100, max = 663) after the trimming described above.

#### Item evaluation

Seven items had item–rest correlations of less than 0.10 and required some optimization. This was particularly true for three items with slightly negative correlations. They were dropped. This did not have much impact on reliability (omega total = 0.77; ICC2k = 0.73).

### Lextale-Esp

#### Descriptive statistics and reliability analysis

Performance on the Lextale test was calculated as the percentage of correct responses to words minus the percentage of false alarms to pseudowords. So, for example, a person with 50 yes responses to words and two yes responses to nonwords would receive a score of 50/60 − 2/30 = 0.77. The mean score obtained was 0.80 (*SD* = 0.12, min = 0.50, max = 1.00, *N* = 213). Because of its multifactorial composition (consisting of words and nonwords), reliability was calculated with the split-half correlation between odd and even items, corrected for length attenuation. It yielded an estimate of *r* = 0.75.

### Correlations between tests

In the previous sections, we analyzed each test on the basis of the participants who performed well on the test. In this section, we examine how well the tests correlated with each other. For this, we limited the data to the 161 participants for whom we had information on all tests (see the section on data trimming). We present the data both for the three StuVoc tests as translated from English, and then for the new selection of two 37-item tests we created. Several aspects are noteworthy.

A first notable observation is that the Lextale scores demonstrated a correlation of about 0.4 with the vocabulary tests based on meaning recognition (multiple-choice questions). This is less than expected considering the reliability of the tests (0.75) and indicates that the two formats measure partly different skills. The divergence of StuVoc-Esp and Lextale-Esp (based on meaning and form recognition, respectively) is also visible in the correlations with the general knowledge test, where Lextale does significantly worse than the tests based on meaning recognition. For example, the correlation between Lextale and GK is significantly lower than the correlation between Lextale and the 74 selected items (*z* = − 3.21, *p* < 0.01). In contrast, the correlation between reading comprehension and Lextale is only slightly lower than the correlations with the new vocabulary tests. These correlations are of similar magnitude as the ones observed in Vermeiren et al., (2023, Study 5): about *r* = 0.3.

A second notable aspect in Table 2 is that the selection of items on the basis of factor analysis and IRT analysis made little difference in the correlations with the other tests. The test with 74 selected items did not do much better than the initial translations, despite the larger number of items. Similarly, the simple translation tests do as well as the two tests with 37 selected items. The test that stands out a bit is the translation of StuVoc3. In Vermeiren et al. (2023), this was a test mainly containing unfamiliar words for familiar experiences, which turned out to be too easy for the population tested. In the present study, we see that the Spanish translation of StuVoc3 is not much easier than translations of StuVoc1 and StuVoc2, but resulted in reduced variability (the *SD* of test 3 is lower than that of test 1 and test 2). This shows up in the correlations with other tests, which are generally lower for test 3, except for the correlation with reading comprehension, which is the highest. We will return to this unexpected observation after Study 2.

**Table 2:**
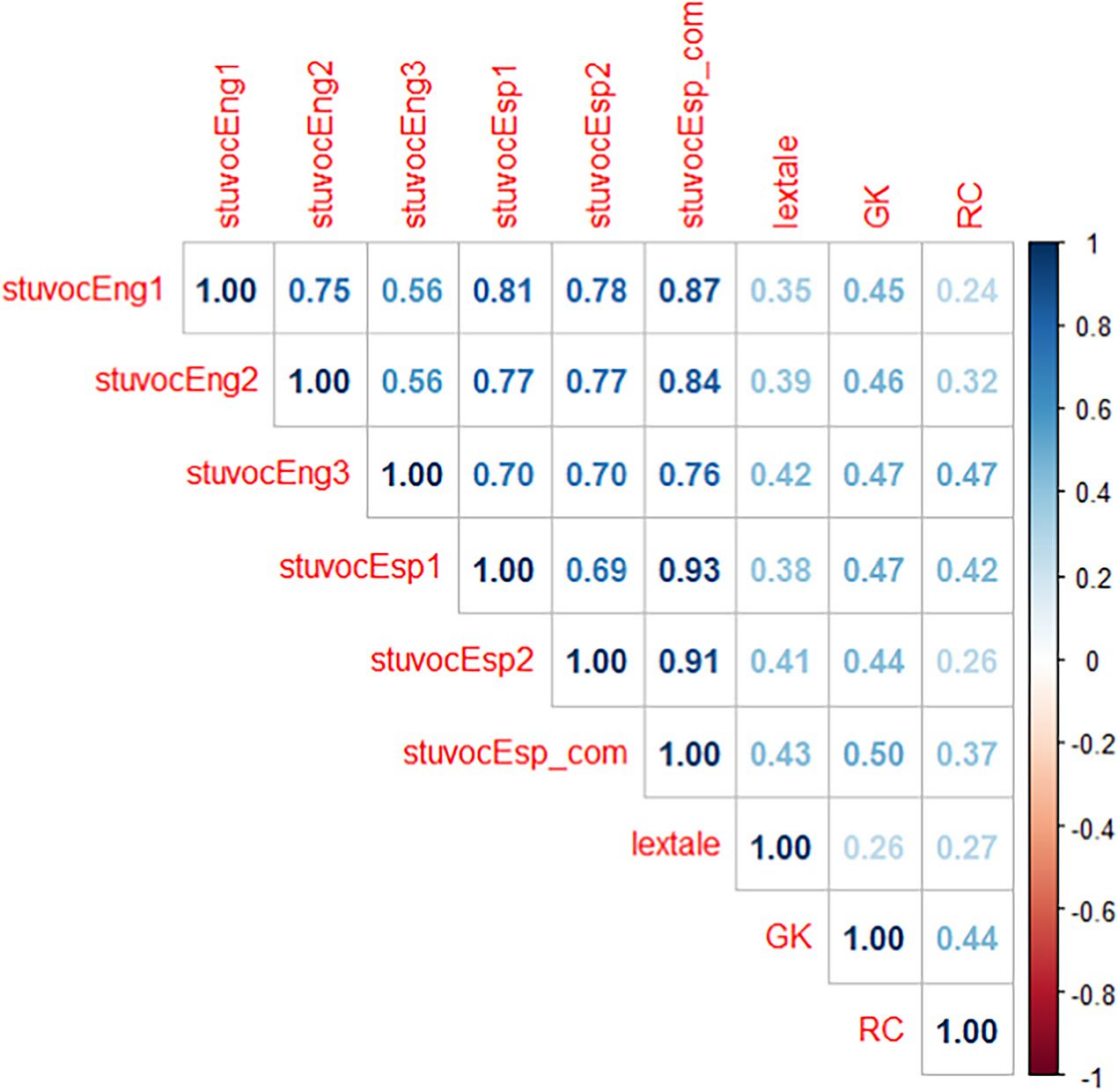
Pearson correlations between participants’ tests scores (*n* = 161)

*Note.* Correlations above *r* =.2 are significant at the.01 level, two-tailed

## Discussion

Study 1 had two main goals: (1) to create a useful Spanish vocabulary test for undergraduate students and educated adults, and (2) to investigate what gains could be made with item translation when creating vocabulary tests for a population with restricted range. Research by Vermeiren et al. (2023) found that it is difficult to select words that reliably differentiate between students with high proficiency and those with lower proficiency, which is understandable given that students have a vocabulary of 40,000 words on average in their native language. Two secondary goals of this study were to determine (3) how well the new StuVoc-Esp test would correlate with two validation criteria, and (4) how well StuVoc-Esp would correlate with Lextale-Esp, another Spanish vocabulary test based on word form recognition rather than meaning recognition.

As can be seen in Table 2, the findings with respect to the two main aims are largely positive. Whereas Vermeiren et al. (2023) needed five studies to build their materials, we were able to obtain equivalent results with a single translation study on the basis of their stimuli. The reliability of the translated tests and correlations between the Spanish tests were very similar to those reported by Vermeiren et al. (2023) for the original English tests. This was true for the vocabulary test(s), but also for the general knowledge test and the reading comprehension test. The last one must not be underestimated. Vermeiren et al. (2023) tried out four different published English reading comprehension tests and observed that none had reliability above 0.6. In four improvements, they managed to increase the reliability of one test to 0.65, and it looks like we may have made some further advancement in the present translation (omega total = 0.77; only 3/42 items doing poorly).

Based on the data we obtained, we selected two vocabulary tests with 37 items, which we thought were the best possible, also considering differences in word knowledge between Spain and Latin America. As can be seen in Table 4 at the end of the article, some words had very high accuracy rates (approaching 100%). We decided to keep them for two reasons: (1) these items are likely to be of interest when the test is given to less proficient Spanish speakers, and (2) easy items are good as control items in online studies to detect careless responding (cf. the control items we added to the vocabulary tests).

A danger in the data in Table 2 is that the findings may be biased by our item selection. We compiled two new StuVoc-Esp tests, which were subsequently correlated with general knowledge and reading comprehension. Although we compiled the tests without looking at the correlations with the other tests, our procedure may have resulted in some overfitting, so that the results found in a new, independent study may be less good than the data presented in Study 1 (Brysbaert, 2024). There were also the concerns that the intercorrelations between the tests in Study 1 were based on only 161 participants, who mainly came from Latin America, whereas we want the test to be useful in Spain as well.

To tackle the issues head-on, we decided it would be good to run a second, cross-validation study, in which the two StuVoc-Esp tests from Table 2 were presented with all other (improved) tests to a new group of participants from Spain.

## Study 2

Study 2 was a cross-validation test to see how well the two StuVoc tests would perform in a new group of participants.

### Method

#### Participants

In total, 235 people (145 female, 86 male, 4 indicating other gender; mean age = 30.37) were tested. Data collection was initiated with students at the University of Salamanca, who were compensated for their participation with course credit (*n* = 91, 78 women, 12 men, 1 indicated as other; mean age = 20.1, mean range = 18–41). This group was included to make sure that our test works with university students, a group often used in psycholinguistics research. Because the sample was smaller than 200 (Brysbaert, 2024) and homogeneous (mainly women coming from a single university), extra participants were recruited via Prolific, who were paid £8.5 for their participation. For this group, we did not impose an upper age limit, so the age was higher (*n* = 144, 67 women, 74 men, 3 indicated as other; mean age = 36.8; range = 19–68). Vocabulary increases with age, and this effect is as large as the effect of education level (Brysbaert et al., 2016; Keuleers et al., 2015). Time for completion of the study varied among participants, with an average of 65 min.

To ensure that data were only collected from native Spanish speakers from Spain, several criteria were applied (native speaker, fluent in Spanish, born in Spain, and Spanish nationality). Participants who had a first language other than Spanish (*n* = 4), who lived in a different country or had a different nationality (*n* = 8), or who had already participated in Study 1 on Prolific (*n* = 3) were excluded. Thus, data from 220 participants were available (mean age = 30.67, range = 18–68, 131 female, 85 male, 4 reported other gender). No further participants had to be excluded due to careless responding (see below).

Of the 220 included participants, 103 were students (46.8%), 92 were working (41.8%), and 25 were both studying and working (11.36%). Participants show heterogeneity in their educational level, ranging from compulsory education (*n* = 6) and high school (*n* = 75), to professional bachelor's degree (*n* = 19), academic bachelor's degree (*n* = 76), master’s degree (*n* = 35), and PhD levels (*n* = 9). Two thirds of participants (*n* = 139, 63.18%) reported having completed a post-secondary, non-compulsory educational program. The majority of participants (*n* = 199, 90.45%) reported speaking one or more languages other than Spanish (190 English, 47 French, 17 German, 9 Basque, 22 Catalan, 18 Galician, 8 Italian, 4 Japanese, 2 Portuguese, 7 other languages).

#### Materials

All materials used for validation in this second study can be found at https://osf.io/c8n2x/ (folder: Tests available for research). Materials were based on Study 1, with minor adjustments based on the item analyses in Study 1, described as follows.

#### Vocabulary tests (StuVoc1-Esp, StuVoc2)

In Study 1, we selected two sets of 37 words based on performance across the Spanish and Latin American participants and on prevalence scores in SPALEX (Aguasvivas et al., 2018). As each test included at least three easy words (higher than 98% accuracy in Study 1), no additional control items were included.

#### General knowledge tests (GK1, GK2)

We excluded the 10 items with poor response profiles in Study 1, leaving us with a shortened, 55-item version of the general knowledge test (GK1).

Because a reviewer of Study 1 wondered why we had not simply made use of the normed items in the database of Spanish general knowledge questions compiled by Buades-Sitjar et al. (2021), we decided to add such a test as GK2. GK1 was developed to make the items vocabulary-light, in order to test knowledge other than names of concepts. However, it can be argued that a general knowledge test also asking about words (e.g., Of what color is cerulean a tonality? Blue/Yellow/Red/Green) could be an even more informative test of crystallized intelligence. To avoid bias in the selection process, the questions were ordered from easiest to most difficult, and a uniform distribution of 53 items was selected between 98% accuracy and 30% (thus above the 25% chance level), excluding those already presented in GK1.

#### Reading comprehension (RC) test

We used the same RC test as in Study 1 but made an effort to find better answer alternatives for the three questions with suboptimal performance in Study 1.

#### Lextale-Esp

The same version of the test as in Study 1 was used in this validation study.

### Procedure

All the tests were administered in LimeSurvey as in Study 1. Participants received the same informed consent, instructions, and demographic questions, and the same order of tests, namely Lextale-Esp, StuVoc1-Esp, StuVoc2-Esp, reading comprehension, and the general knowledge tests GK1 and GK2. The participants were first recruited from university students and then via Prolific (see the “Participants” section for more information). As in Study 1, all tests took place online.

## Results

### Data trimming

Data trimming was conducted in the same way as for Study 1. Prior to analysis, the mean completion time was computed for each test, and responses with a duration 2.5 *SD*s above or below the average time or with performance close to chance level (50% for Lextale, 25% for the other tests) were discarded. This represented 5% of the data in the vocabulary tests (thus leaving 209 out of 220 initial participants), 2.72% in Lextale-Esp (214 out of 220 participants), 2.30% in the general knowledge test 1 (212 out of 217 initial participants), and 2.31% in the general knowledge test 2 (211 out of 216 initial participants). For the reading comprehension test, words read per minute were calculated per text, and those texts with reading rates higher than 668 words per minute or lower than 100 words per minute were considered outliers and excluded from subsequent analyses (12.38% of the texts presented). No other participants had to be excluded because of poor performance on easy vocabulary items. Four easy items in each StuVoc-Esp test were answered correctly by all remaining participants (StuVoc 1: *alardear*, *rechupete*, *lentejuela*, *acaramelar*; StuVoc 2: *desmoronar*, *sincero*, *manutención*, *trueque*) and could not be included for the calculation of the omega total. Altogether, 196 out of 220 participants completed all tests well and could be included in the correlation analyses.

### Test descriptive statistics

As in Study 1, analyses were run with R software, including descriptive analysis and reliability analysis (omega total and ICC2k), with the R package psych (Revelle, 2021). Tests with omega reliability above 0.75 were considered good (Koo & Li, 2016). All data and R code can be found at https://osf.io/c8n2x/files/; folder Study 2).

Table 3 summarizes the findings. First and foremost, they show that the findings with the StuVoc-Esp1 and StuVoc-Esp2 are stable. Means, standard deviations, and reliability indices are very similar. The tests show a correlation of *r* = 0.76 with each other (*n* = 209). All other tests are also in line with Study 1, although performance on Lextale-Esp was higher and showed evidence for a ceiling effect. The new general knowledge test, GK2, was very similar to knowledge test GK1.

**Table 3:** Information about all tests used in Study 2

	Mean (*SD*)	Min	Max	Omega total	ICCk2
StuVoc-Esp 1 (37 items)	31.8 (3.64)	20	37	0.78	0.70
StuVoc-Esp 2 (37 items)	31.3 (3.80)	18	37	0.77	0.71
Combined (74 items)	63.1 (6.96)	43	74	0.87	0.83
Lextale-Esp (90 items)	0.91 (0.10)	.50	1.00	0.70 ^a^	
GK1 (55 items)	36.4 (7.42)	16	55	0.85	0.80
GK2 (53 items)	35.8 (8.29)	14	53	0.88	0.85
RC (42 items)	0.78 (0.12)	0.33	1.00	0.78	0.71

^a^ Split-half correlation

### Correlations between tests

Table 4 shows the Pearson correlations between tests for participants with data on all tests (*n* = 196). All correlations are very much in line with what was expected from Study 1 and are even higher. Again, the new StuVoc-Esp tests correlate more highly with the general knowledge tests and the reading comprehension test than Lextale. Also interesting is the high correlation between the general knowledge tests and reading comprehension (see also Study 1).

**Table 4:**
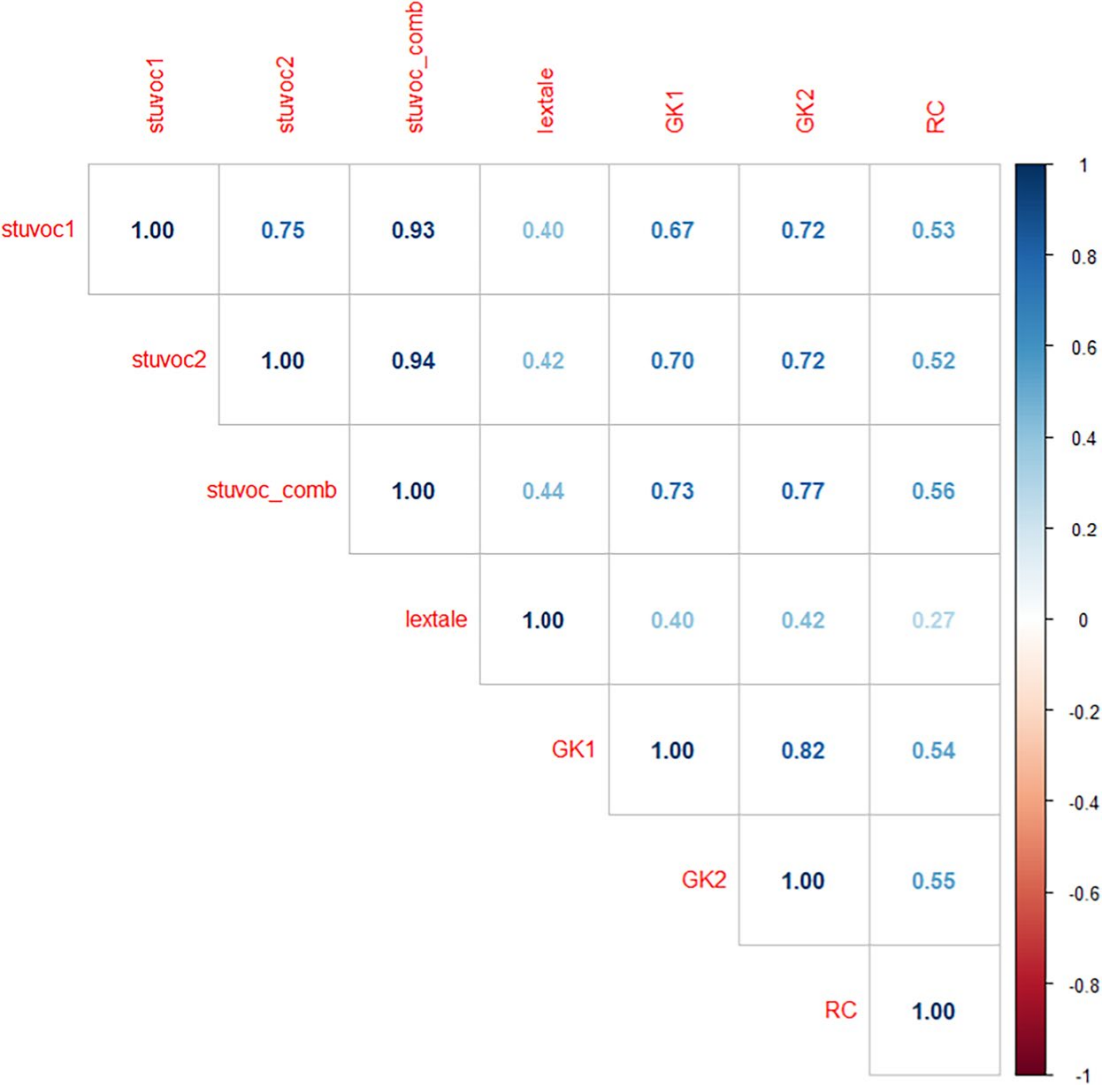
Pearson correlations between participants’ tests scores (based on data from 196 participants)

**Fig. 6:**
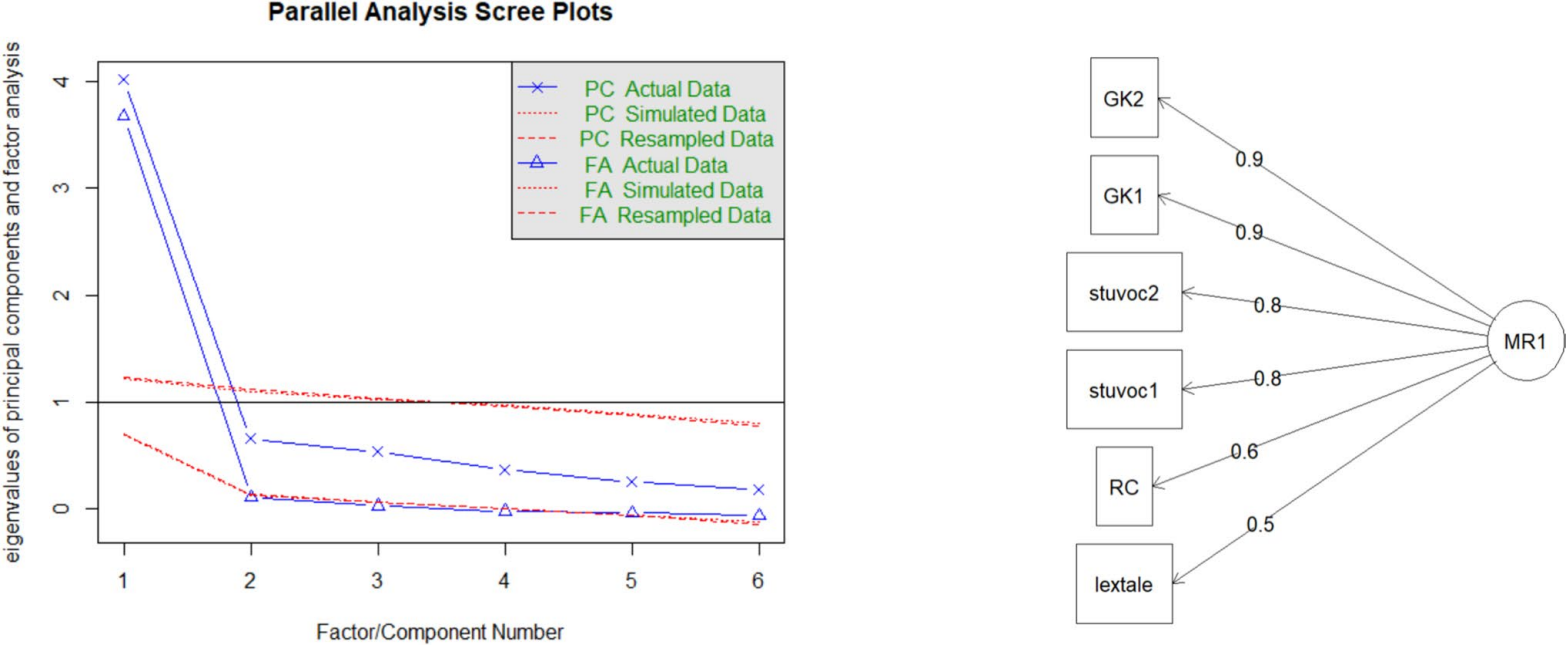
Principal components and factor analysis. *Note*. Parallel analysis scree plot (left). The *x*-axis represents the number of extracted factors or principal components, and the *y*-axis represents the eigenvalues of principal components and factor analysis, which indicate the amount of variance explained by each factor/component. Factor loading diagram (right). The strength of the relationship between each observed variable and a single latent factor (MR1) is displayed. Principal components and factor analysis both indicated that a unifactorial model best described the intercorrelations between the tests

To determine how best to group the tests, we used factor analysis. The scree plot indicated that a single factor accounted for the correlations observed (Fig. 6, left part). The loadings were highest for the general knowledge tests and the StuVoc-Esp tests (Fig. 6, right part).

## Discussion

The cross-validation study confirmed the usefulness of the StuVoc-Esp1 and StuVoc-Esp2 vocabulary tests compiled in Study 1. It also showed that general knowledge tests are a good predictor of reading comprehension. Lextale-Esp performed less well, but that may be partly because the test was too easy for the sample of participants tested.

## General discussion

In this article we investigated how much mileage could be obtained by using translation in the construction of language tests for high-performing participants. The results were largely positive: Although some item loss was unavoidable, we were able to create two good vocabulary tests, one general knowledge test and one reading comprehension test, basically in a single study (Study 2 was added to make sure we were not making unsubstantiated claims). With respect to the general knowledge test, Study 2 showed that a translated test was as good as a test based directly on normed items in the target language (Spanish).

These findings open the possibility for developing equivalent tests in different languages, at least as long as the languages are as closely linked as English and Spanish. Further research will have to indicate whether the same is possible for more distant languages/cultures. The reason why translation works is that it is very difficult to find the right materials for native adult speakers. There are no schoolbooks to take inspiration from, easy materials are likely to be known by everyone, and difficult materials risk not being known by anyone (hence not making much difference between individuals). This makes it very difficult to find a sweet spot if one must start from scratch. Translations from another language with more information then are a better approach than blindly trying out materials based on one’s own intuitions.

To avoid confusion, we want to state explicitly that translation only works for item generation. It does not automatically promise test reliability and validity. Knowledge of the latter requires at least one validation study as described in Study 1. Two validation studies are even better if item selection involves several steps and criteria (as shown in Study 2).

One might object because, for the vocabulary tests, we benefited from the availability of Spanish word prevalence norms (Aguasvivas et al., 2018). This allowed us to better match the Spanish translations with the English originals. Such information is not available in many other languages. Fortunately, however, collecting prevalence information for a small number of words can be easily done. Researchers only need to create some nonwords and present a list of interesting translation candidates together with the nonwords to some 20–40 participants from the target population, asking them which words they know. The percentage of participants who say yes (and do not exhibit an overly large bias to saying yes to nonwords) provides the word prevalence information.

Another factor that helped was that we could start with more items than strictly needed. Item loss is inevitable in translation, because items can be language- and culture-specific, so that their difficulty and/or discrimination ability are not the same between the original version and the translated version. If there are not enough items in the source language, it may still be possible to use that language as an initial guess if it contains much more information about the stimuli than the target language. English is the best resourced language. It may be easier to search for materials in this language and translate them rather than search blindly in the language without resources.

Our main aim was to make a good Spanish vocabulary test for educated, adult native speakers. We were able to compile a test with 74 items that has high reliability and correlates well with general knowledge and reading comprehension. At the same time, the data showed that not much extra precision is lost from 74 to 37 items (Tables 2 and 4). Researchers worried about time requirements can therefore limit themselves to StuVoc-Esp1 or StuVoc-Esp2, use both tests at different moments in the testing, or make an adaptive test in which words are ordered in five batches of 12 items and one batch of 14 items from easy to hard. Participants are asked to start with batch four and they get more difficult batches as long as they respond correctly to more than four items. Participants who have less than 9/12 items correct on batch four also get batch 3 and, if needed, batches 2 and 1.

In two studies, the StuVoc tests predicted reading comprehension better than Lextale. This is consistent with the conclusion drawn by McLean et al. (2020), who found that although the yes/no format of Lextale scores well on reliability, it predicts reading proficiency less well than a meaning recognition test. These authors found even better prediction with recall tests, where participants must give an answer rather than select it from a list. Zhang and Zhang (2020) reached a similar conclusion based on a meta-analysis. On the other hand, Siegelman et al. (2024) reported correlations around 0.5 between Lextale and reading comprehension in both a large group of native English speakers and large groups of second-language speakers, which was not much lower than scores based on a meaning recognition test (around 0.6), although this study also pointed to a higher correlation between the StuVoc format and reading comprehension than between the Lextale format and reading comprehension.

Still related to the prediction of reading comprehension scores, we saw some evidence for a higher correlation with the translation of StuVoc-Eng3 than with the translations of StuVoc-Eng1 and StuVoc-Eng2. Vermeiren et al. (2023) saw a similar pattern in their results with the English tests. One possible interpretation of this finding is that knowledge of some words is more helpful than others for understanding texts. If so, further mileage may be possible by searching for the most informative words and whether these words differ as a function of the text being read. In the present studies, the texts were expository encyclopedia-type paragraphs, with a rather high information load. It may be worthwhile to try out some reading comprehension tests based on more exciting fiction and see whether the same types of words are predictive.

We also saw good predictive value of the general knowledge tests we used. To some extent this is not a surprise given the expository nature of the reading materials. Participants with background knowledge may retain more from such texts than readers without background knowledge. Or, alternatively, readers who remember much from what they read may build up a larger knowledge base than readers who remember less (both factors could also reinforce each other). Our findings, together with those of Vermeiren et al. (2023), certainly point to the usefulness of including a general knowledge test in reading research. Again, it will be interesting to see whether this is still true for the prediction of reading comprehension with fiction texts.

## Availability of the materials

We strongly believe that language research requires access to freely available tests which researchers can build upon (in different languages). Therefore, we make all materials from the present study available at https://osf.io/c8n2x/; see also Vermeiren et al., 2023, for the English materials). They are free to use for scientific research (not for commercial purposes) if properly attributed. It is our hope that other researchers will find them useful and, equally importantly, will use them to further improve the tests available in Spanish. Therefore, we additionally present the data from the item analyses we did, so that authors can see which items work well and which could use improvement.

Although most vocabulary items we translated seemed to work, we recommend colleagues use the tests with 37 items we selected. These items largely measure a single factor and are well distributed across the full range of difficulty (which should make the test useful for somewhat less proficient and somewhat more proficient participants, although ideally this is tested before the test is used). For many purposes, a test with 37 items will do, and there are diminishing returns for longer tests. However, the availability of two tests allows researchers to combine them in ways they see fit. In Table 5 we show the target words that were selected for the test, the mean accuracy rates in Study 1 (Acc1) and 2 (Acc2), and the item–rest correlations in both studies (Item-cor1 and Item-cor2).

**Table 5:** Target words selected for StuVoc-Esp1 and StuVoc-Esp2, together with information about mean accuracy and item–rest correlation in Study 1 and Study 2

StuVoc-Esp1					StuVoc-Esp2				
Target word	Acc_1	Acc_2	Item_cor1	Item_cor2	Target word	Acc1	Acc2	Item_cor1	Item_cor2
compost	0.89	0.86	0.23	0.36	paradigma	0.64	0.76	0.09	0.33
cuatrero	0.54	0.55	0.36	0.51	reprimir	0.99	0.98	0.16	0.06
errático	0.75	0.80	0.21	0.48	desmoronar	0.94	1.00	0.29	NA
excretar	0.58	0.70	0.27	0.22	alfanje	0.45	0.52	0.30	0.43
umbral	0.65	0.87	0.14	−0.01	jaranear	0.84	0.96	0.20	0.07
impío	0.59	0.64	0.49	0.53	fervor	0.83	0.89	0.32	0.33
alardear	0.97	1.00	0.37	NA	obstinado	0.87	0.82	0.36	0.37
decrepito	0.92	0.94	0.13	0.27	funesto	0.88	0.98	0.24	0.18
disidencia	0.77	0.88	0.29	0.45	oblea	0.86	0.90	0.30	0.19
rechupete	0.93	1.00	0.28	NA	campesinado	0.92	0.98	0.28	0.07
tumulto	0.83	0.92	0.26	0.21	marga	0.36	0.38	0.26	0.35
jovial	0.92	0.96	0.22	0.25	asignar	0.88	0.85	0.11	0.23
lúgubre	0.91	0.97	0.47	0.20	puritano	0.93	0.97	0.16	0.25
sinvergüenza	0.84	0.90	0.24	0.38	retro	0.95	0.96	0.21	0.05
pragmática	0.64	0.88	0.23	0.32	sarcófago	0.68	0.83	0.20	0.06
auge	0.96	0.99	0.31	0.00	miniatura	0.95	0.95	0.12	0.13
saliente	0.46	0.41	0.17	0.19	eludir	0.93	0.98	0.15	0.23
arcada	0.82	0.99	0.39	−0.02	fulguroso	0.72	0.76	0.27	0.49
arrasar	0.87	0.95	0.19	0.16	escalofrío	0.99	1.00	0.10	−0.07
cafeína	0.96	0.98	0.11	−0.06	balandra	0.66	0.72	0.22	0.39
rublo	0.83	0.82	0.21	0.46	omnipresente	0.98	1.00	0.26	0.14
ostentoso	0.92	0.95	0.42	0.27	taxón	0.57	0.64	0.28	0.33
homogéneo	0.98	0.99	0.13	0.16	fractura	0.99	0.98	0.14	0.08
lentejuela	0.99	1.00	0.11	NA	marsupial	0.85	0.91	0.19	0.30
rendija	0.81	1.00	0.45	0.13	sincero	0.98	1.00	0.09	NA
maligno	1.00	0.99	NA	0.10	inefable	0.59	0.59	0.23	0.15
acaramelar	0.98	1.00	0.27	NA	yola	0.67	0.56	0.25	0.33
resina	0.99	1.00	0.19	−0.08	empalar	0.79	0.75	0.35	0.51
manta	0.99	0.96	0.17	0.20	yunque	0.89	0.94	0.37	0.43
flancos	0.85	0.90	0.38	0.29	manutención	0.96	1.00	0.47	NA
desistir	0.68	0.44	0.18	0.25	sagaz	0.72	0.77	0.21	0.50
afincar	0.87	0.98	0.41	0.16	pergamino	0.91	0.97	0.38	0.29
virar	0.87	0.89	0.25	0.47	ahondar	0.87	0.97	0.22	0.27
augurio	0.68	0.67	0.34	0.33	chapado	0.51	0.62	0.24	0.35
devoto	0.91	0.98	0.31	0.25	trueque	1.00	1.00	NA	NA
ablución	0.34	0.45	0.38	0.54	tiento	0.41	0.52	0.17	0.28
atril	0.51	0.56	0.16	0.16	raído	0.87	0.98	0.35	0.12

In addition to the vocabulary test, we also provide access to the general knowledge test and the reading comprehension test that we used. The full tests are given, together with item information, so that researchers can improve poorly performing items (and consequently the overall quality of the tests).

## Limitations

Future studies may consider overcoming some of the limitations of the current study. First, despite the advantages of online data collection in terms of efficiency and convenience, researchers may prefer better control of participant performance during data collection. Efforts were made to ensure participant cooperation during the tasks, but this cannot be controlled to the same extent as in laboratory research. Another way to improve control could be to videotape participants during data collection or to use specialized software to obtain information about window switching (Diedenhofen & Musch, 2017). We did not notice anything improper in our data collection that could not be addressed in the data analysis, but we understand that researchers may want more control.

Another limitation of the present study is that participant information is based entirely on self-report and showed a large variety in participant characteristics. For instance, the participants came from various places, which are likely to have country-specific Spanish words. Care was taken to avoid issues of country-specific language use by selecting words generally known and used in Spanish. Importantly, such variability is also positive, since it increases the chances that the present test will work in different settings, not only in a laboratory and with a particular group of participants. Nonetheless, other researchers may first want to repeat our study with a more restricted group (i.e., the students from their own university) to see to what extent the findings apply to this specific population. By making the test items available, we want to facilitate this type of cross-validation. Future studies can also deepen the contribution of region-specific differences in vocabulary knowledge and student characteristics.

## Data Availability

The datasets generated during and/or analyzed during the current study are available in the OSF repository at https://osf.io/c8n2x/.

## Footnotes

1 Language proficiency is also a big topic in children’s L1 acquisition. However, researchers here usually make use of commercial vocabulary tests and do not develop many tests themselves.

2 The translation of all tests and the collection and analysis of the data from Study 1 took approximately 6 weeks.

3 Reliability of a test determines the standard error of measurement (*SEM*). When reliability is 0, *SEM* is the same as the standard deviation observed in the test scores (*SD*), meaning that the test score provides no extra information about individual differences. When reliability is 1, *SEM* is 0, and participants are expected to achieve identical values on parallel tests. More generally, the equation connecting *SEM* to *SD* is *SEM* = *SD* × sqrt(1 − reliability). Thus, reliability of.75 means that *SEM* =.50 *SD*; reliability of.9 means that *SEM* =.31 *SD*.

4 We thank an anonymous reviewer for pointing out this requirement. For instance, we dropped the target words *chochin* and *curruca*, because they refer to birds typically found in Europe.

## Code availability

Materials and analysis code are available at https://osf.io/c8n2x/. None of the reported studies was preregistered.
